# The Potential of MicroRNAs as Clinical Biomarkers to Aid Ovarian Cancer Diagnosis and Treatment

**DOI:** 10.3390/genes13112054

**Published:** 2022-11-07

**Authors:** Molly Davies, Matthew G. Davey, Nicola Miller

**Affiliations:** Department of Surgery, The Lambe Institute for Translational Research, University of Galway, H91 YR71 Galway, Ireland

**Keywords:** ovarian cancer, miRNA, non-coding RNA, personalised medicine, biomarkers

## Abstract

Ovarian cancer is a commonly diagnosed malignancy in women. When diagnosed at an early stage, survival outcomes are favourable for the vast majority, with up to 90% of ovarian cancer patients being free of disease at 5 years follow-up. Unfortunately, ovarian cancer is typically diagnosed at an advanced stage due to the majority of patients remaining asymptomatic until the cancer has metastasised, resulting in poor outcomes for the majority. While the molecular era has facilitated the subclassification of the disease into distinct clinical subtypes, ovarian cancer remains managed and treated as a single disease entity. MicroRNAs (miRNAs) are small (19–25 nucleotides), endogenous molecules which are integral to regulating gene expression. Aberrant miRNA expression profiles have been described in several cancers, and have been implicated to be useful biomarkers which may aid cancer diagnostics and treatment. Several preliminary studies have identified candidate tumour suppressor and oncogenic miRNAs which may be involved in the development and progression of ovarian cancer, highlighting their candidacy as oncological biomarkers; understanding the mechanisms by which these miRNAs regulate the key processes involved in oncogenesis can improve our overall understanding of cancer development and identify novel biomarkers and therapeutic targets. This review highlights the potential role of miRNAs which may be utilised to aid diagnosis, estimate prognosis and enhance therapeutic strategies in the management of primary ovarian cancer.

## 1. Introduction

### 1.1. Ovarian Cancer: A Heterogenous Disease

Ovarian cancer is among the most commonly diagnosed gynaecological malignancies worldwide and is associated with high rates of mortality [1,2,3,4]. The poor oncological and survival outcomes observed in patients diagnosed with ovarian cancer is best explained by the subclinical growth of such tumours causing a delayed onset of non-specific symptoms [1,4], which is reflected in late diagnoses, when the cancer is at an advanced stage. Recent data from the National Cancer Registry Ireland illustrates that ovarian cancer is currently the fourth most common cause of cancer-related mortality in women in the Republic of Ireland [5].

The molecular era has progressed to recognise that ovarian cancer is a heterogenous disease made up of several behaviourally distinct intrinsic biological subtypes [6,7]. Such subtypes may be distinguished and characterised based on several parameters, including the anatomical origin of the cancer, the clinical behaviour of the disease, the biomolecular tumour profile, and the overall genetic instability of the disease [4]. The most common form of the disease is epithelial ovarian cancer, followed by germ cell and sex-cord-stromal ovarian, which together account for approximately 5% of cases [3]. Epithelial ovarian cancer can be further substratified into four pathologically distinct primary subtypes, which include serous (low-grade or high-grade), endometroid, clear cell and mucinous carcinoma [3]. In addition, malignant epithelial ovarian tumours may be subdivided into type I and type II tumour types, where type I represents the more indolent, less aggressive clinical subtype, and type II tumours behave aggressively and are typically associated with poor anticipated survival outcomes [4]. Typically, serous, endometrioid, clear cell and mucinous ovarian cancers which are low-grade are considered to represent type I disease, while high-grade serous tumours are classified to be type II disease. Importantly, high-grade serous ovarian cancer (HGSOC) is the most common form of the disease [6,8] and typically responds poorly to conventional therapeutic strategies, leading the disease to be responsible for up to 80% of all ovarian cancer-related deaths [8]. Notwithstanding our appreciation for the differences in the aforementioned ovarian cancer subtypes, management of the disease is currently homogenous irrespective of biomolecular differences in cancers, relying on an antiquated, untargeted approach, with the exception of poly adenosine diphosphate-ribose polymerase (PARP) inhibition in the setting of BRCA alteration carriers. At present, conventional first line treatment relies on the blunderbuss approach of combined taxane-based and platinum-based chemotherapies, irrespective of the basic clinicopathological and molecular differences within the disease [3]. Therefore, translation research efforts are now focused on applying basic sciences to the clinical paradigm of ovarian cancer diagnostics and therapeutics to facilitate earlier diagnosis of the disease.

### 1.2. Biomarkers and Ovarian Cancer

A biomarker, a portmanteau of ‘biological marker’, is a characteristic that is objectively appraised to provide an indication of normal biological processes, pathological processes, or pharmacological responses to a therapeutic intervention [9]. Biomarkers are endogenous molecules which are detectable, measurable and quantifiable to indicate a state of disease. Diagnostic biomarkers can aid the earlier detection of disease while predictive and prognostic biomarkers can facilitate the personalisation of treatment strategies [10]. Commonly evaluated examples of biomarkers relevant to malignancy include proteins and genetic material, including deoxyribonucleic acids (DNA), ribonucleic acids (RNA) and genetic alteration status [11,12,13]. Biomarker discovery is a well-established yet exciting field within the realm of translational research, and the development of novel oncological biomarkers remains at the forefront of translation research priorities. Such efforts aim to use non-invasive means to decipher novel diagnostic strategies, inform patient-specific prognosis, and monitor disease progression in the setting of metastatic disease [10].

Several biomarkers are currently used in clinical practice to aid cancer diagnosis and management, such as Carcinoembryonic Antigen (CEA) in colorectal cancer, Bladder Tumour Antigen (BTA) in bladder cancer, Prostate Specific Antigen (PSA) in prostate cancer [14], Cancer Antigen 125 (CA125) in ovarian cancer, and the BRCA1/2 mutation in breast and ovarian cancer [15] (Table 1). CA125 is a protein which is may be elevated to a detectable level in the circulation of up to 80% of patients diagnosed with epithelial ovarian cancer [1]. CA125 may be isolated from patient serum samples and quantified using antibody-based immunoassays, such as enzyme-linked immunosorbent assay (ELISA) [16]. While CA125 is a useful diagnostic biomarker, it also has utility in gauging therapeutic response to conventional treatment, with levels of the biomarker being measured and evaluated during treatment, to assess patient response to treatment. Notwithstanding, CA125 is limited by moderate sensitivity levels in the setting of early disease [1,17], unreliable measurements during certain physiological processes such as menstruation [18], and varying acceptable measurement levels in patients of a certain age, race, and body mass index [19]. Therefore, the identification of novel minimally invasive biomarkers to aid ovarian cancer diagnostics, inform prognosis and to gauge therapeutic response to treatment are imperative to improve the anticipated oncological outcomes for these patients.

### 1.3. MicroRNAs as Cancer Biomarkers

**Figure 1:**
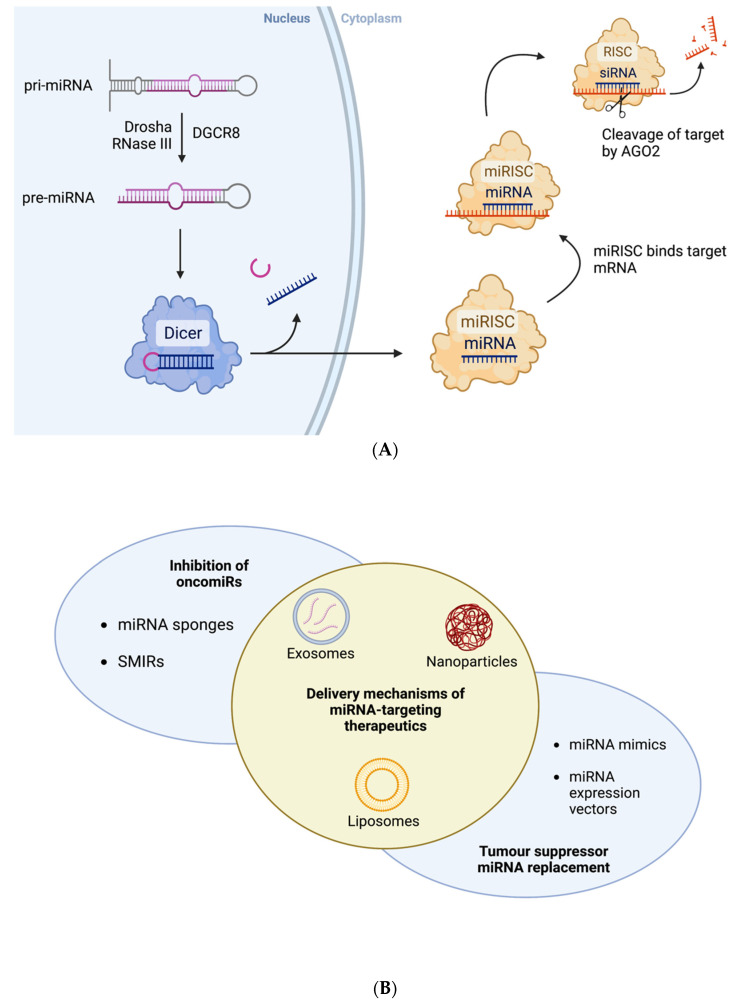
(**A**) Schematic of normal biogenesis and function of miRNAs. (**B**) Summary of miRNA-based therapeutic strategies and mechanisms of delivery to cells. Created with BioRender.com.

MicroRNAs (miRNAs) are a contemporary class of short non-coding RNA (sncRNA). The molecules are approximately 19 to 25 nucleotides in length and have been illustrated to play an integral role in regulating gene expression [23,24]. MiRNAs were first described by Lee et al. in 1993 when studying developmental timing of *Caenorhabditis elegans* [25], and the scientific understanding of the role of miRNA has exponentially grown in recent years, with aberrant miRNA expression profiles now understood to correlate with several diverse pathological processes, including oncogenesis [26,27,28,29]. MiRNAs regulate gene expression at a post-transcriptional level by binding to the 3′ or 5′ untranslated regions of target messenger RNA (mRNA), hindering mRNA expression through degradation or translation inhibition. All miRNAs are initially synthesised within the nucleus as primary miRNA transcripts (pri-miRNA). The pri-miRNA is usually processed by DGCR8 and Drosha RNase III to form precursor-miRNA (pre-miRNA), which is transported to the cytoplasm. Pre-miRNA molecules are subject to further processing by the Dicer RNase III endonuclease to form a mature miRNA molecule [23,24]. Once the mature miRNA is synthesised it can be incorporated into the miRNA-indued silencing complex (miRISC). The miRNA acts as a guide strand as its sequence is complementary to that of its target mRNA. A protein in the miRISC, Argonaute 2 endonuclease (AGO2), cleaves the target. It is by this mechanism that miRNAs normally function to silence genetic expression [23,24] (Figure 1A).

Aberrant miRNA expression profiles have been commonly observed within cancer cells, highlighting their potential as biomarkers and therapeutic targets in malignancy [2,11,24]. Thus, translational research efforts have focused on understanding the biomolecular mechanisms underpinning miRNA dysregulation and their impact upon oncogenesis. Identifying the idiosyncratic miRNA expression pattern specific to each cancer subtype has also been investigated to decipher novel molecular subtypes [30]. Therefore, it is plausible that miRNA expression profiles may potentially play several roles in ovarian cancer treatment, from expediting diagnosis, to designing more targeted treatment regimens, to monitoring patient-specific response to therapeutics [11,31].

MiRNAs can be divided up into two fundamental classes based on their target genes and oncological implications on tumour development [24]. Tumour suppressors act to silence genetic information implicated in uncontrolled proliferation and subsequently halt cancer progression [32]. Intuitively, tumour suppressor miRNAs are typically downregulated in cancer cells, triggering potentially uncontested tumourigenesis. The second class, oncogenic miRNAs (typically described as oncogenes or oncomiRs), are molecules which promote cancer development through increasing expression of cancer inducing genes, thereby contributing to oncogenesis. Conversely to tumour suppressor miRNAs, oncomiRs are classically upregulated in the setting of malignancy [32].

MiRNAs have been extracted and amplified from Formalin Fixed Paraffin Embedded (FFPE) tissue, blood (including serum and plasma), saliva, urine, and other bodily fluids in amounts which are suitably quantifiable for analysis [33]. MiRNAs may be amplified relatively simply and inexpensively using quantitative reverse transcription polymerase chain reaction (qRT-PCR), leading to this technique being the most commonly utilised method for amplifying complementary DNA (cDNA) synthesised from extracted RNA [11]. The expression levels of target miRNAs can be assessed using qRT-PCR by comparing the cycle threshold (C_T_) values generated by each target of interest (i.e., miRNAs), which are quantified relative to endogenous controls [34,35]. Other miRNA profiling methods include miRNA microarray and RNA sequencing, however these methods are limited by decreased sensitivity and the inability to determine absolute quantification of miRNAs. There is also a high cost associated with RNA sequencing, as well as complex data analysis [11,33].

### 1.4. MiRNAs as Therapeutic Agents

The increased understanding of the regulatory roles of miRNAs presents these endogenous molecules as therapeutic tools with significant potential in the targeted treatment of cancer [36]. Several strategies have been developed to interfere with the expression of specific oncomiRs and tumour suppressor miRNAs (Figure 1B). Sandwich RNAi inhibition involves simultaneous targeting of an oncomiR by a combination of siRNA- and miRNA-based technologies [36]. This dual targeting has demonstrated enhanced therapeutic efficiency. Nishimura et al. targeted the expression of EphA2, an oncogenic protein expressed in ovarian cancer, using siRNA and miR-520d-3p which resulted in robust depletion of the protein levels [37]. MiRNA sponges are synthetic oligonucleotides which have been designed to have high affinity for target miRNA molecules [38]. MiRNA sponges act as competitive inhibitors with specific oncomiR silencing capabilities. Small molecule miRNA inhibitors (SMIRs) have been designed to directly bind to target miRNAs thereby interfering with their normal mechanisms of gene silencing [39]. Conversely, RNA restoration therapy has also been explored as a potential therapeutic strategy. MiRNA mimics, synthetic RNA duplexes which are designed to resemble endogenous miRNAs, have been transiently transfected into cells and have been successful in upregulating target gene expression [40]. Exosomes, nanoparticles, lentivirus and plasmid expression vectors are some of the delivery mechanisms explored for transduction of these miRNA-based therapeutics to target cells [36] (Figure 1B).

### 1.5. Tumour Suppressor MiRNAs Associated with Ovarian Cancer

As previously outlined, tumour suppressors act to silence genetic information and subsequently attempt to prevent tumour progression. Several tumour suppressor miRNAs have been identified and their roles in the development of several cancers are beginning to be unravelled. For example, miRNA expression profiling has demonstrated that the miR-15/16 cluster and the miR-34 family are commonly under expressed in colorectal and prostate carcinoma [41]. Moreover, miR-101 has been illustrated to be involved in several biological processes associated with cancer development including tumour proliferation, angiogenesis and metastasis [42]. MiR-101 has been implicated to be typically downregulated in cancerous tissues, and plays a role in silencing multiple target oncogenes, such as *SOX2* [43], *DNMT3A* [44] and *EZH2* [45]. EZH2 is a catalytic subunit of polycomb repressive complex 2 (PRC2) and plays a major role in regulating gene expression by catalysing the trimethylation of H3 lysine 27 (H3K27) [42,45]. Epithelial ovarian cancer cell viability has been shown to be dependent on EZH2 expression, and therefore, EZH2 inhibition, is a promising targeted therapeutic strategy [46], highlighting the potential of miR-101 in the treatment of epithelial ovarian cancer. This dependency however is seen in certain mutational contexts, further emphasising the importance of understanding ovarian cancer heterogeneity [46]. A recent investigation by Wei et al. evaluated the impact of miR-101 levels in ovarian cancer cells and illustrated further evidence supporting the tumour suppressor role of miR-101 in malignancy [47]. Furthermore, miR-101 levels were significantly decreased in ovarian cancer cells when compared to the normal ovarian cell lines. Moreover, this study highlighted *PTEN*, a well-known tumour suppressor gene (TSG) [48], which supports the viability of ovarian cancer cells, as illustrated by the potential to use this tumour suppressor miRNA to target *PTEN*, providing further insight into miR-101 as a potential therapeutic target for ovarian cancer [47]. Li et al. investigated the tumour suppressor role of miR-584 by analysing miRNA expression levels from ovarian cancer patient tissue and tumour-associated normal (TAN) tissue, as well as in ovarian cancer cell lines [49]. The results illustrated miR-584 as being significantly downregulated in ovarian cancer tissue and cells. Using the ovarian cancer cell lines, a potential gene target of miR-584 could be identified to further explore the role of miR-584 in cancer development. Alongside this, using patient tumour tissue samples allowed for the investigation of the prognostic role of miR-584, and it was determined that increased miR-584 expression levels correlated with improved overall survival in ovarian cancer patients [49]. A recent study be Liu et al. sought to evaluate the role and biological mechanisms of miR-27b-5p regulation in ovarian cancer [50]. Expression levels of the tumour suppressor miRNA were again analysed using patient tumour and TAN samples, as well as in ovarian cancer cell lines. The authors identified that miR-27b-5p was significantly downregulated in ovarian cancer cases, and that it may have a role in the development and progression of ovarian cancer, potentially by targeting the *CXCL1* gene. Furthermore, miR-27b-5p also shows potential for use as a prognostic biomarker and therapeutic target in ovarian cancer treatment, as the variation in expression levels seen in tumour samples was correlated to tumour stage, metastasis and overall survival [50]. Table 2 summarises some recent findings in investigations into tumour suppressor miRNAs which have shown potential as diagnostic and/or prognostic biomarkers in ovarian cancer, as well as novel therapeutic targets.

Members of the let-7 family of miRNAs play essential roles in regulating development and cellular differentiation [58,59,60]. Therefore, it is unsurprising that the aberrant expression of let-7 miRNAs is associated with cancer. There are ten members of the let-7 family, namely, let-7a, let-7b, let-7c, let-7d, let-7e, let-7f, let-7g, let-7i, miR-98 and miR-202 [60], and dysregulation of several let-7 miRNAs has been reported in a range of cancer types [60,61,62,63]. In the majority of these cases, expression of let-7 miRNAs is decreased, which has been shown to result in elevated levels of target oncogenic proteins such as RAS, Myc, and LIN28 [58]. Let-7g was previously shown to be significantly downregulated in the serum and tumour tissue of epithelial ovarian cancer patients [55]. The exact role of let-7 miRNAs in ovarian cancer development however remains ambiguous, as overexpression of let-7 family members has also been demonstrated in the setting of malignancy [58]. Nonetheless, this family of miRNAs has significant potential as diagnostic and prognostic biomarkers. In addition, there has been accumulating evidence that let-7 miRNAs could be utilised in potential therapeutic strategies. For example, Cai et al. identified that let-7e expression was significantly reduced in cisplatin-resistant human epithelial ovarian cancer cells [64]. By reintroducing let-7e, the cells became partially resensitised to cisplatin chemotherapeutics. The role of each let-7 miRNA family member should be explored within specific subtypes of ovarian cancer, as their roles in carcinogenesis could vary between the heterogenous forms of the disease.

### 1.6. OncomiRs Associated with Ovarian Cancer

Song et al. illustrated mir-221 to be aberrantly overexpressed in several types of human cancers [65], however, its role in ovarian cancer is yet to be accurately evaluated. In an attempt to explore this, Li et al. evaluated miR-221 expression levels between ovarian cancer tissue and TAN, as well as between ovarian cancer cell lines and normal ovarian cells [66]. The results showed that miR-221 was significantly upregulated in ovarian cancer cases. Furthermore, it was noted that miR-221 could be informative of tumour size, stage and invasion, and that higher expression of the oncomiR was associated with a poorer prognosis and oncological outcomes. Overall, higher miR-221 expression was correlated with decreased disease-free survival and overall survival [66].

Humphries et al. coherently summarise the role of the miR-200 family in cancer [67]. The highly conserved miR-200 family is made up of miR-200a, miR-200b, miR-200c, miR-141 and miR-429, and is frequently studied in cancer biology. A meta-analysis of miRNA expression signatures in epithelial ovarian cancer identified miR-200a and miR-200c as the two most highly dysregulated miRNAs in this particular cancer type [68]. Mir-200a and miR-200c were significantly upregulated in epithelial ovarian cancer cases. This overexpression of miR-200a/c is correlated with improved outcomes of epithelial ovarian cancer patients [68]. The sensitivity and specificity of miR-200a/c was also analysed, which provided further support for their potential use as diagnostic biomarkers. Although preliminary, findings such as these warrant further investigation as oncomiRs could serve as useful diagnostic and prognostic biomarkers for the early detection of ovarian cancer. Meng et al. investigated the expression levels of a panel of seven circulating miRNAs with a known association with cancer in the serum of epithelial ovarian cancer patients [69]. Among these, miR-25 and miR-93 were significantly downregulated, while miR-7 and miR-429 were significantly upregulated in the cancer patient samples compared to the serum of the healthy controls. Furthermore, a positive correlation between miR-429 and CA-125 levels was observed in ovarian cancer patients, highlighting its potential as a diagnostic and prognostic ovarian cancer biomarker [69]. In another study, a panel of ten miRNAs were found to have potential diagnostic relevance in ovarian cancer [70]. Among these, miR-96-5p, miR-182, miR183, miR-141-5p, miR200a/b/c and miR429 were significantly upregulated in ovarian cancer tissue samples compared normal ovarian tissue, demonstrating some overlap with other studies, as summarised in Table 3.

### 1.7. Using MiRNAs to Differentiate between Female Cancers

Previous researchers have focused their efforts into identifying novel diagnostic biomarkers which can aid the early detection of cancer, as cancer diagnosis at an early stage is key to ensuring successful treatment and enhancing patient outcomes. This is particularly important in cases of ovarian malignancies, as symptoms are relatively non-specific, with presentations usually reserved until the cancer has progressed to an advanced stage. The management of metastatic ovarian cancer is challenging to the oncologist as the disease typically becomes more resistant to treatment. Thus, the application of miRNA panels (rather than just a single miRNA type) may yield more informative information in aiding cancer diagnostics. In a preliminary study by Hirschfeld et al., the expression levels of 25 clinically relevant miRNAs were analysed in breast, endometrial and ovarian cancer cells [75]. It was outlined that by comparing miRNA expression patterns across the different cell types, differentiation between cancer subtypes could be successfully achieved. The most significant findings relating to ovarian cancer miRNAs were that let-7b, miR-21, and miRNAs from the miR-30 family were upregulated in ovarian cancer cells relative to breast cancer cells. Furthermore, it was detailed that miRNA expression patterns could potentially distinguish ovarian cancer cells from endometrial cancer cells. MiR-92a, miR-106a and miR-200b were upregulated in endometrial cancer cells compared to ovarian, and conversely miR-222 was upregulated in ovarian cancer cells compared to endometrial cancer cells [75]. Hui et al. illustrated that miRNA signatures successfully differentiated uterine serous carcinomas from ovarian serous carcinomas [76]. Notwithstanding, if serous carcinoma is diagnosed at an advanced stage, distinction between primary and metastatic tumours often proves challenging [76]. Such factors have clinically significant implications as they effect decision-making in terms of treatment regimens for the patient. The translation of miRNA expression analysis into clinical practice could potentially aid the diagnosis of metastatic cancer.

### 1.8. Using MiRNAs to Determine Ovarian Cancer Subtype

As previously outlined, the theory that ovarian cancer is a homogenous disease is antiquated [6,7]. Furthermore, the prescription of generic treatment regimens in ‘blanket’ fashion for all patients with ovarian cancer opposes the dogma of precision oncology [77]. Identifying miRNA signatures specific to ovarian cancer subtypes would aid in clinical diagnosis and have a direct impact on choice of treatment. Efforts have been made to identify miRNA expression patterns which are unique to ovarian cancer subtype in an attempt to identify novel diagnostic and prognostic biomarkers. In one particular study, miRNAs of the miR-192/215 family (miR-192, miR-194, miR-215), were found to be significantly upregulated in mucinous carcinoma tissue, and downregulated in each other subtype [78]. The exact biological pathways of the miR-192/215 family in ovarian cancer are yet to be uncovered. Yanaihara et al. identified miR-9 as a potential diagnostic biomarker and therapeutic target for ovarian clear cell carcinoma [79]. In comparison to HGSOC, significantly higher expression of miR-9 was observed in clear cell carcinoma cells. Another study highlighted the potential of using miRNAs to differentiate between different subtypes of epithelial ovarian cancer [80]. The main finding of this study was that miR-510 expression levels varied significantly across high-grade serous, clear cell and low-grade serous ovarian cancer tissue. These results illustrate the importance of miRNA expression levels to highlight variety between the different subtypes of the disease, supporting the concept that ovarian cancer is not a homogenous disease. Inversely, ovarian cancer should be divided into clinical and therapeutically distinct molecular subtypes with targeted therapies specific to inhibit the biological activity of the tumour, while minimising the treatment associated toxicities associated with robust chemotherapy prescription. These differential miRNA expression patterns between female cancer types and between ovarian cancer subtypes are summarised in Table 4.

## 2. Limitations

A major challenge in identifying reliable, meaningful diagnostic and prognostic biomarkers in ovarian cancer research is the lack of reproducibility of results. Although there are some relatively consistent findings in the literature, a significant proportion of results remain to be supported by evidence and validated. This research is still at a preliminary stage, and although initial studies are important in order to identify candidate miRNA targets for investigation, the small sample sizes often bring the meaningfulness and reproducibility of findings into question. Methodological variations between studies are evident, from the sample storage and preparation stages to the subsequent analysis stages. For example, RNA isolation protocols and PCR conditions commonly vary between studies. In an attempt to limit inconsistencies, efforts should be made to create and optimise a standard protocol for miRNA analysis in clinical samples. Another important confounding factor in miRNA investigations using the qRT-PCR method is the inclusion of a suitable reference miRNA, known to be stably expressed, called an endogenous control [33,81,82]. Suitable endogenous controls for the normalisation of miRNA expression levels in ovarian cancer are to be validated. U6 is used as the endogenous control in most miRNA quantification studies, however, its reliability has been questioned recently as unstable expression of U6 has been reported [81,82]. Bignotti et al. investigated the reliability of eleven candidate endogenous controls for the normalisation of miRNA quantification studies in HGSOC. SNORD48 was identified as a potentially reliable control, followed by miR-191-5p [82]. Other candidates have recently been identified for epithelial ovarian cancer, namely, miR-106b-3p and miR-92b-5p [81].

Experimental design factors are within the control of researchers and working towards standardising protocols to offset inconsistencies which may arise between studies is possible. However, a number of factors remain outside of the control of researchers, including tumour heterogeneity, and external and lifestyle elements which can influence miRNA expression patterns. Lifestyle choices such as diet and smoking habits have been shown to influence human epigenetics and miRNA expression [83,84]. Factors such as patient age and gender have been shown to directly impact miRNA expression [85,86], and could potentially be accountable for a lack of reproducibility between studies. This influence can unfortunately reduce the power of results from miRNA expression studies, which hinders the translation of miRNAs as biomarkers and therapeutic strategies into clinical practice. In an attempt to limit these inconsistencies researchers could try to carry out miRNA expression investigations within better-defined cohorts. For example, when working with patient samples, and indeed with controls, it may prove useful to work with samples from those a certain age range, where resources allow.

## 3. Future Perspective

This review has successfully identified candidate miRNAs which may have potential roles in the development, molecular substratification and treatment of ovarian cancer. Due to these studies being predominantly preclinical studies, these data may be considered preliminary with potential for translation into clinical practice should results be ratified. Notwithstanding, further limitations of these studies include their predominantly small sample sizes and inherent selection biases, meaning further scientific investigation is required at a larger scale before these candidate biomarkers are clinically applied to ovarian cancer management. One of the most pertinent clinical issues in the management of ovarian cancer is the earlier detection of the disease, which could in theory be translated into improved oncological outcomes [87]. MiRNA expression profiling across the different tumour stages may be useful to fully establish the role of these molecules as diagnostic biomarkers, particularly when other circulatory biomarkers such as CEA have moderate sensitivity levels in the setting of early stage disease [88]. As previously outlined, CA125 is limited as circulatory levels of the biomarker are typically detectable only in patients with advanced-stage ovarian cancer [1,17]. Thus, identifying circulatory miRNAs with aberrant expression profiles in the setting of stage I disease would have potential to revolutionise earlier diagnosis of the disease, and by proxy, improving anticipated survival outcomes. Furthermore, Talaat et al. demonstrated that miR-21 had improved sensitivity, specificity and accuracy as an ovarian cancer diagnostic biomarker compared to CA125 [89]. Incorporating miRNAs alongside protein biomarkers such as CA125 in ovarian cancer diagnostic tests has the potential to improve their reliability. Importantly, miRNA expression profiling can be performed causing minimal discomfort to the patient as blood, urine and saliva samples have been shown to contain sufficient quantities of miRNAs for downstream analysis [21,90,91,92,93].

The field of precision oncology is continuously evolving to aid the optimal design of treatment regimens for cancer patients. Alongside their potential as biomarkers, miRNA expression profiles have also been shown to correlate with patient response to cancer treatment. As summarised in the recent review by Davey et al., breast cancer miRNA signatures are potentially informative of how likely a patient will respond or develop resistance to neoadjuvant chemotherapy [94]. As previously mentioned, patients diagnosed with metastatic ovarian cancer tend to have a poor prognosis as the cancer cells often develop a resistance to chemotherapy. Comparing the differential expression patterns of miRNAs between patients who respond effectively to treatment and patients experiencing chemoresistance could result in the identification of predictive and prognostic biomarkers and in turn a better understanding of the potential role played by miRNAs in developing drug resistance. This could be utilised to direct therapeutic decision-making and design patient-specific treatment strategies. Yu at al. showed that miR-206 was upregulated in chemoresistant epithelial ovarian cancer tissue, compared to tissue from patients who experienced a clinical complete response [95]. Zhang et al. [96] showed that the expression pattern of miR-181c negatively correlates with that of glucose-regulated protein 78 (GRP78), a protein which is known to be expressed in cancer and contributes to resistance to platinum-taxane chemotherapeutics [96,97]. Using miRNA mimics, miR-181c levels were restored in ovarian cancer cells, resulting in increased sensitivity of the ovarian cancer cells to Paclitaxel. This study suggests targeting GRP78-associated pathways as a potential strategy overcome the major challenge of chemoresistance in ovarian cancer management [96]. This highlights the importance of further research into the field of precision oncology and miRNA expression profiling as there is significant clinical potential, however, translating this research from bench to bedside remains a challenge.

Identification of ovarian cancer-associated miRNAs is still in its infancy, and efforts should be made in order to validate candidate biomarkers and better understand their roles in carcinogenesis. Understanding the roles of the key tumour suppressors and oncomiRs that drive ovarian cancer development is a fundamental part of improving early detection of the disease and personalising treatment for patients. Emphasis should continue to be placed on evaluating miRNA signatures of the heterogenous disease in order to improve patient survival rates and overall quality of life.

## Figures and Tables

**Table 1:** Clinically established cancer biomarkers.

Cancer Type	Biomarker	Reference
Breast	ER	[20]
	PgR	[20]
	HER2	[20]
	Ki-67	[10]
	BRCA1/2 mutation	[15]
Colorectal	CEA	[14]
Bladder	BTA	[21]
	NMP22	[21]
	BladderCheck Test	[21]
Lung	EarlyCDT-Lung	[22]
	CEA	[22]
	CYFRA21-1	[22]
	ProGRP	[22]
Prostate	PSA	[14]
Ovarian	CA125	[1]
	BRCA1/2 mutation	[15]
	HE4	[1]

ER: estrogen receptor; PgR: progesterone receptor; HER2: human epidermal growth factor receptor 2; CEA: Carcinoembryonic Antigen; BTA: Bladder Tumour Antigen; NMP22: Nuclear Matrix Protein 22; CYFRA21-1: cytokeratin 19 fragment; ProGRP: Progastrin-releasing peptide; PSA: Prostate Specific Antigen; CA125: Cancer Antigen 125; HE4: Human Epididymis Protein 4.

**Table 2:** MiRNAs downregulated in ovarian cancer.

Year	Tissue/Cell Type	N	miRNA	Reference
2021	SW626, SKOV3, OVACAR3, PA1	N/A	miR-101	Wei et al. [47]
2020	A2780cp, A2780s, SKOV3, CAOV3	N/A	miR-193b	Li et al. [51]
2020	Tumour & TAN	45	miR-27b-5p	Liu et al. [50]
2020	Tumour & TAN	31	miR-584	Yang et al. [49]
2020	SKOV3	N/A	miR-155-5p	Ysrafil et al. [52]
2018	Blood, OVCAR3	8	miR-195	Chen [53]
2016	Tumour, SKOV3, OVCAR-429	20	miR-125a, miR-125b	Lee et al. [54]
2019	Tumour & TAN, serum, OVAR3, HEY-A8	17	let-7g	Biamonte et al. [55]
2021	Tumour & TAN, SKOV3, A2780	10	miR-585-3p	Lu et al. [56]
2021	Serum	30	miR-193a-5p	Zhang et al. [57]

N = number of tissue samples, TAN = tumour-associated normal.

**Table 3:** MiRNAs upregulated in ovarian cancer.

Year	Tissue/Cell Type	N	miRNA	Reference
2021	Plasma EVs	34	miR-4732-5p	Liu et al. [71]
2020	Tumour & TAN, CAOV3, SKOV3	11	miR-141, miR-200a, miR-7, miR-203, miR-18a, miR-93, miR-106a, miR-20a, miR-19a	Wahab et al. [72]
2019	Serum EVs	48	miR-93, miR-145, miR-200c	Kim et al. [73]
2017	Tumour & TAN, A2780, OVCAR3, SKOV3, 3AO	63	miR-221	Li et al. [66]
2016	Tumour & TAN, SKOV3, OV56, A2780, COV362, EFO21, OV90	145	miR-760	Liao et al. [74]
2014	Tumour & TAN	487	miR-96-5p, miR-182, miR-183, miR-141-5p, miR-200a, miR-200b, miR-200c, miR-429	Wang et al. [70]
2015	Serum	180	miR-7, miR-429	Meng et al. [69]

N = number of tissue samples, TAN = tumour-associated normal, EV = extracellular vesicle.

**Table 4:** Variation in miRNA expression between (i) ovarian cancer and other female cancers and (ii) ovarian cancer subtypes.

Study	Differential miRNA Expression Patterns
Hirschfeld et al. [75]	Let-7b, miR-21 and miR-30 family members upregulated in ovarian cancer cells compared to breast cancer cells miR-92a, miR-106a and miR-200b upregulated in endometrial cancer cells compared to ovarian cancer cells miR-222 upregulated in ovarian cancer cells compared to endometrial cancer cells
Hui et al. [76]	Panel of 6 miRNAs (miR-196b, miR-138, miR-146b-5p, miR-9, miR-155, and miR-454) capable of differentiating between serous uterine carcinoma and serous ovarian carcinoma
Agostini et al. [78]	miR-192/215 family members upregulated in mucinous carcinoma tissue
Yanaihara et al. [79]	miR-9 upregulated in clear cell carcinoma cells compared to HGSOC cells
Zhang et al. [80]	miR-510 expression was significantly higher in low-grade serous carcinoma and clear cell carcinoma tissue compared to HGSOC and normal ovarian tissue

## Data Availability

Not applicable.
